# Supplementary material for: A Deep-Learning-Based Method Can Detect Both Common and Rare Genetic Disorders in Fetal Ultrasound
Source: Biomedicines. 2023 Jun 19;11(6):1756. doi: 10.3390/biomedicines11061756 (PMC10295959; doi:10.3390/biomedicines11061756)
Supplement: Supplementary file 1 [file biomedicines-11-01756-s001.zip › biomedicines-2401783-supplementary.pdf]

Supplementary Information

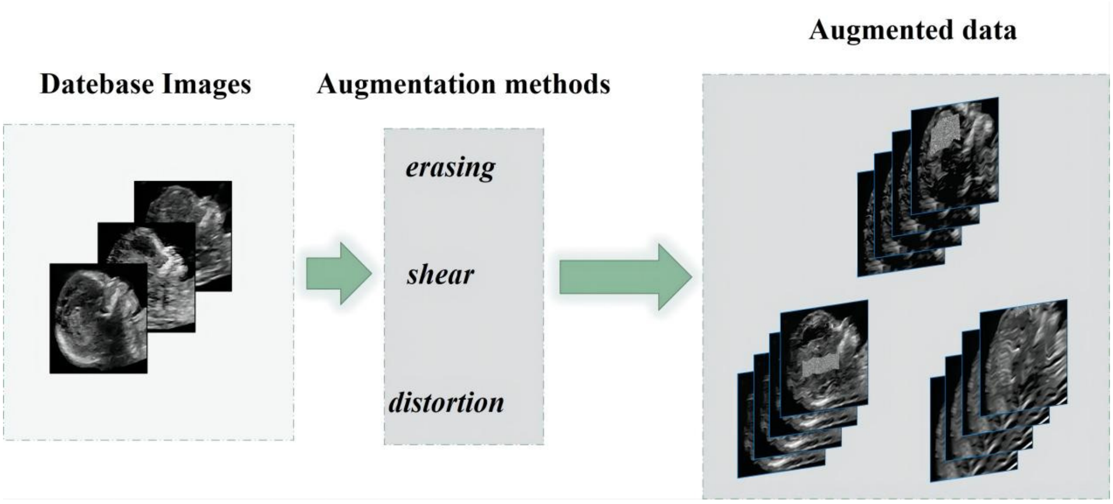

Figure S1. Augmentation Methods: We used distortion, zoom in, tilt, zoom out, crop, and a combination of multiple methods to augment the training date set.

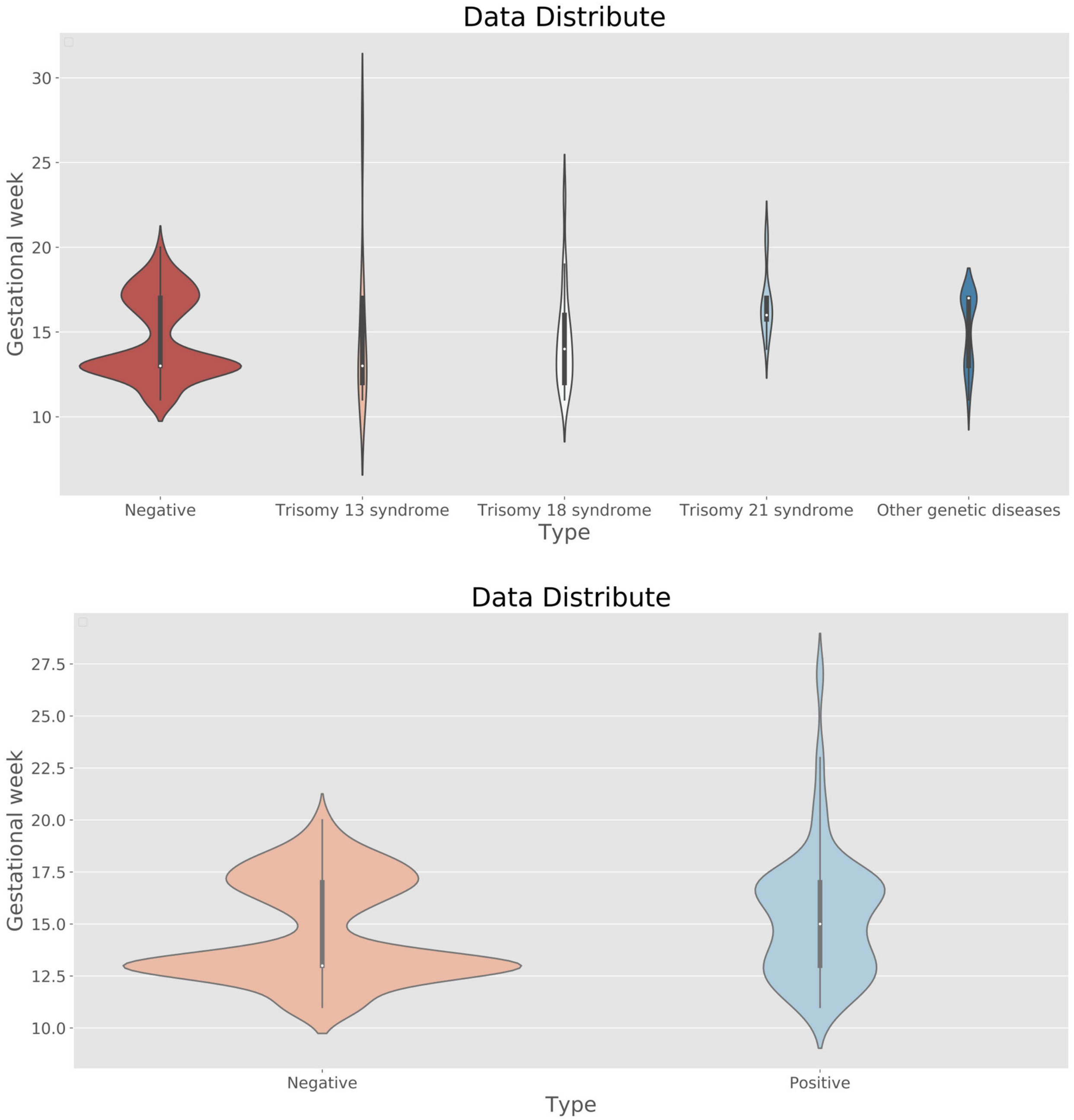

Figure S2. Violin Plot: Gestational age distribution of each group, including negative, positive, Trisomy 21, Trisomy 18, Trisomy 13 and other genetic disease groups.

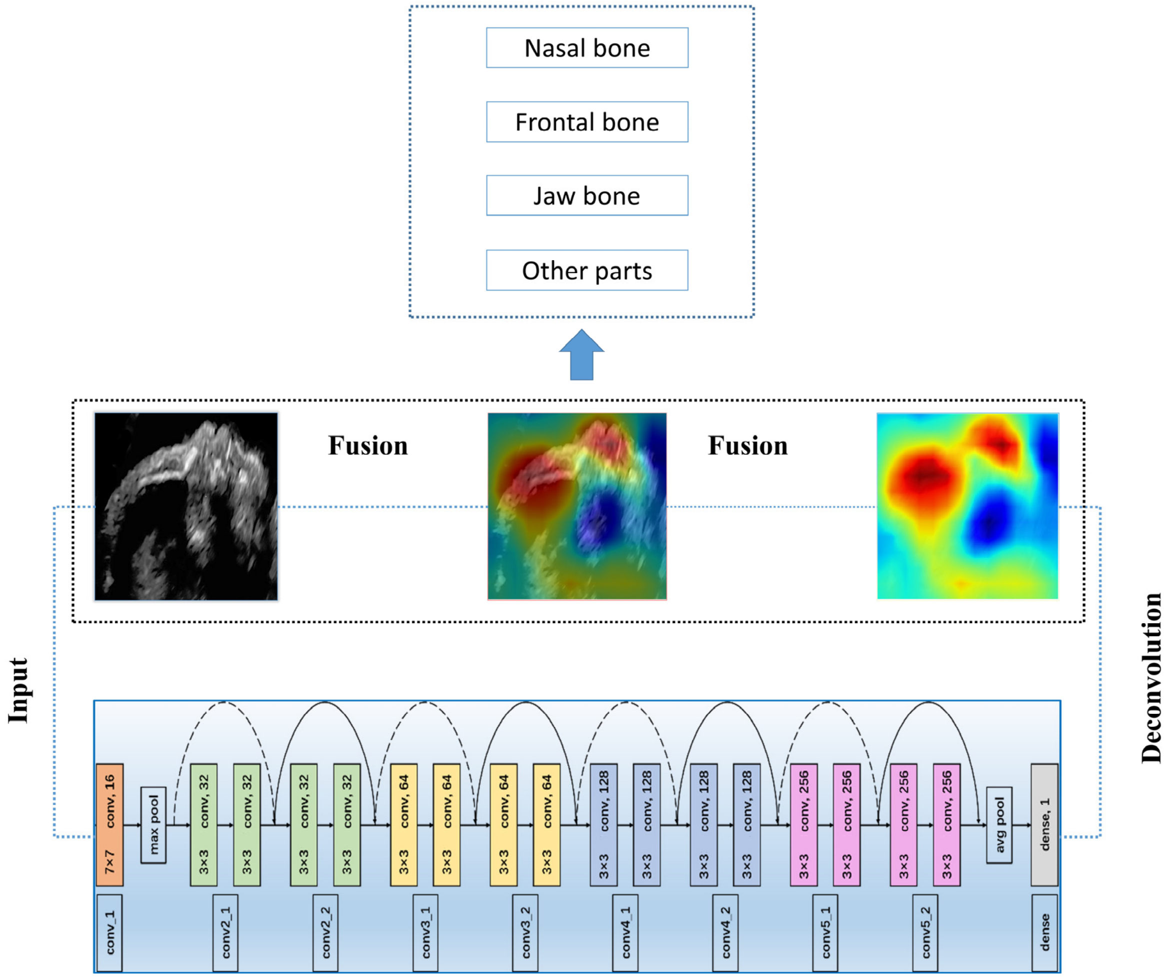

Figure S3. Model structure frame: Model parameters of Pgds-ResNet's and the framework of the interpretable experiment.

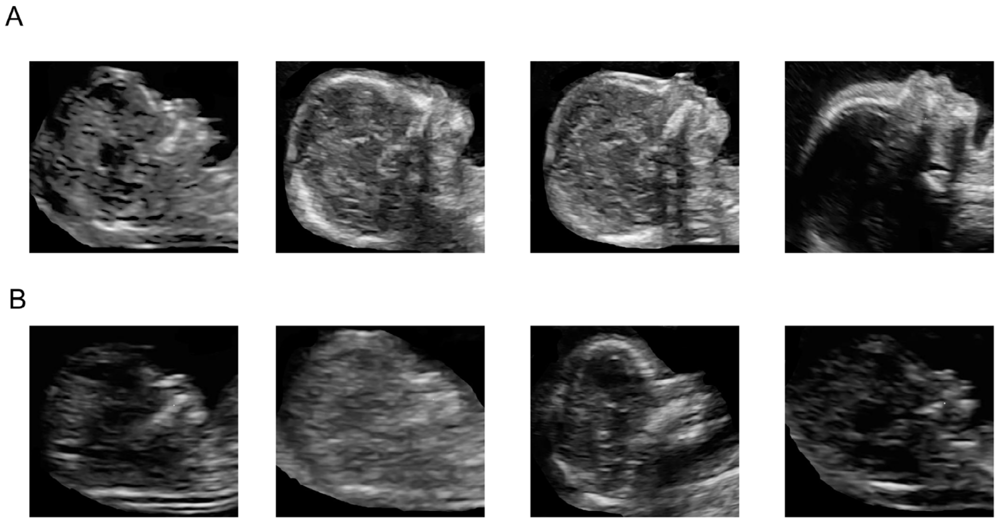

Figure S4. Image quality comparison. A. Good quality. B. Poor quality.

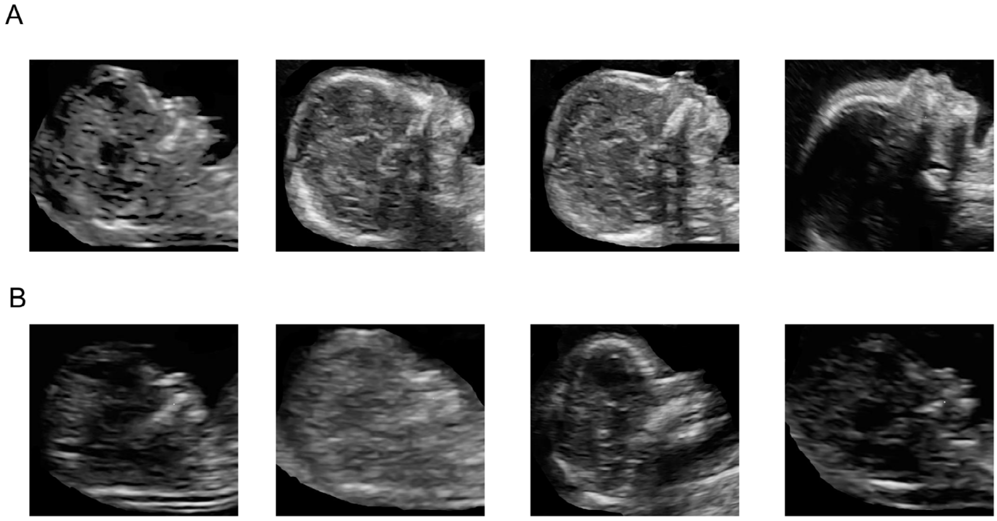

Figure S5. Image quality comparison. A. Good quality. B. Poor quality. In this study, image quality was identified as the main cause of incorrect classification results. The images in B had poor quality, which led to misclassification by the model.
